# Atezolizumab/Bevacizumab vs. Lenvatinib as First-Line Therapy for Unresectable Hepatocellular Carcinoma: A Real-World, Multi-Center Study

**DOI:** 10.3390/cancers14071747

**Published:** 2022-03-29

**Authors:** Beom Kyung Kim, Jaekyung Cheon, Hyeyeong Kim, Beodeul Kang, Yeonjung Ha, Do Young Kim, Seong Gyu Hwang, Young Eun Chon, Hong Jae Chon

**Affiliations:** 1 Department of Internal Medicine, Yonsei University College of Medicine, Seoul 03722, Korea; beomkkim@yuhs.ac (B.K.K.); dyk1025@yuhs.ac (D.Y.K.); 2 Department of Medical Oncology, CHA Bundang Medical Center, CHA University, Seongnam 13496, Korea; cheonjk526@gmail.com (J.C.); wb0707@cha.ac.kr (B.K.); 3 Department of Internal Medicine, Ulsan University Hospital, University of Ulsan College of Medicine, Ulsan 44033, Korea; kimhy@uuh.ulsan.kr; 4 Department of Gastroenterology, CHA Bundang Medical Center, CHA University, Seongnam 13496, Korea; yeonjung.ha@chamc.co.kr (Y.H.); sghwang@cha.ac.kr (S.G.H.)

**Keywords:** atezolizumab, bevacizumab, comparison, hepatocellular carcinoma, lenvatinib

## Abstract

**Simple Summary:**

This study compared the therapeutic efficacy and safety of atezolizumab plus bevacizumab versus lenvatinib as first-line therapy for the treatment of unresectable hepatocellular carcinoma (HCC). A total of 232 patients from three academic hospitals in Korea were included. No significant differences in objective response rate, overall survival, progression-free survival, or the incidence of adverse events were noted. Similar results were obtained after propensity score matching and inverse probability of treatment weighting analyses. This study makes a significant contribution to the literature because it provides the first comparison of lenvatinib and atezolizumab/bevacizumab for the treatment of unresectable HCC in the real-world setting.

**Abstract:**

Lenvatinib (LENV) and atezolizumab/bevacizumab (ATE/BEV) have been approved as first-line regimens for the treatment of unresectable hepatocellular carcinoma (HCC). We aimed to compare their clinical efficacy and safety. Patients receiving ATE/BEV (*n* = 86) or LENV (*n* = 146) as first-line treatment were recruited from three academic hospitals in Korea. Overall survival (OS), progression-free survival (PFS), and radiological response were assessed according to the Response Evaluation Criteria in Solid Tumors. Clinical features of the two groups were balanced through propensity score (PS) matching with a 1:1 ratio and inverse probability of treatment weighting (IPTW) analyses. The median age was 62 years, with male predominance (83.6%). There was no significant difference in the objective response rate between the ATE/BEV and LENV groups (32.6% vs. 31.5%; *p* = 0.868). Neither median OS (not reached vs. 12.8 months; *p* = 0.357) nor PFS (5.7 vs. 6.0 months; *p* = 0.738) was different between ATE/BEV and LENV groups. PS-matched and IPTW analyses yielded comparable results in terms of OS and PFS (all *p* > 0.05). Grade ≥ 3 adverse events occurred in 42.8% and 21.9% of patients in the ATE/BEV and LENV groups, respectively (*p* = 0.141). The two first-line therapy regimens for unresectable HCC had comparable clinical efficacy and safety in real-world practice settings. Further studies with a larger sample size and longer follow-up are needed to validate these results.

## 1. Introduction

Every year, approximately 750,000 patients are diagnosed with hepatocellular carcinoma (HCC), which remains the leading cause of cancer-related death worldwide, especially in the countries of East Asia, including Korea [1,2,3]. Although considerable advances have been made in the surveillance, diagnosis, and treatment of patients with HCC, most cases are unfortunately diagnosed at an advanced stage with a poor prognosis [4,5]. Over the past decade, sorafenib (SOR), an oral multi-kinase inhibitor, has been the only anticancer agent to effectively improve overall survival (OS) [6,7]. Since 2018, lenvatinib (LENV), another oral multi-kinase inhibitor that targets vascular endothelial growth factor (VEGF) receptors, fibroblast growth factor (FGF) receptors, platelet-derived growth factor (PDGF) receptor-α, RET, and KIT, has been used as a first-line treatment option for unresectable HCC [8]. In the REFLECT study, the LENV arm showed a non-inferior OS compared to the SOR arm (median 13.6 vs. 12.3 months), in addition to a significantly improved progression-free survival (PFS; median 7.4 vs. 3.7 months, respectively; *p* < 0.001) and objective response rate (24.1% vs. 9.2%, respectively; *p* < 0.001) [9]. Immunotherapy enhancing host anti-cancer immunity through the blockade of the PD-1/PD-L1 interaction has been established as a major approach for the treatment of various cancer types. In advanced HCC, PD-1 inhibitor monotherapy, such as with nivolumab or pembrolizumab, exhibited clinically meaningful response rates, ranging from 17 to 20% in phase I/II trials [10,11]. However, both agents failed to meet the primary endpoints in the subsequent phase III trials of CHECKMATE-459 and KEYNOTE-240, respectively [12,13]. In contrast, combination therapy of atezolizumab (anti-PD-L1) plus bevacizumab (anti-VEGF-antibody) showed an objective response rate of 36% and a median PFS of 7 months in a phase Ib trial [14]. Based on these promising results, the recent phase III clinical trial, IMbrave 150, showed that the combination of atezolizumab (ATE) plus bevacizumab (BEV) resulted in a significant improvement in the median PFS (6.8 vs. 4.3 months, *p* < 0.001) and OS (13.2 months vs. not reached, *p* < 0.001), when compared to SOR [15]. However, to date, no study has compared the clinical outcomes of two recently approved first-line regimens for unresectable HCC, namely, LENV vs. ATE/BEV. Herein, we performed a multi-center study to compare the clinical efficacy and safety between LENV and ATE/BEV as a first-line regimen for the treatment of patients with unresectable HCC in the real-world setting.

## 2. Materials and Methods

Consecutive patients treated with either LENV or ATE/BEV as first-line systemic therapy for unresectable HCC between August 2019 and July 2021 in three academic hospitals in South Korea (Yonsei University Severance Hospital, CHA Bundang Medical Center, and Ulsan University Hospital) were screened for eligibility. HCC was diagnosed histologically or radiologically in accordance with the latest international guidelines [16,17,18]. The CONSORT diagram is shown in Supplementary Figure S1. The study was reviewed and approved by the Institutional Review Board of Severance Hospital (IRB No. 2021-07-071), Ulsan University Hospital (IRB No. 2021-07-069), and CHA Bundang Medical Center (IRB No. 2021-09-085). Informed consent was waived owing to the retrospective nature of the analyses.

### 2.1. Treatment Regimens

LENV was administered orally, and the dose was determined based on the patient weight: 8 mg/day for patients weighing < 60 kg and 12 mg/day for those weighing ≥ 60 kg. ATE/BEV was administered intravenously with 1200 mg of ATE plus 15 mg/kg of body weight of BEV every 3 weeks.

During the study period, dose modification and treatment interruptions were allowed according to the drug-related toxicity grade, as recommended. In the LENV group, the initial dose was reduced from 12 to 8 mg according to the physicians’ decision. LENV or ATE/BEV was discontinued when unacceptable/serious adverse events (AEs) or tumor progression were observed.

### 2.2. Assessment of Clinical Outcomes

Patients were followed up every 6 to 12 weeks to assess treatment response and safety. Radiological response was assessed according to the Response Evaluation Criteria in Solid Tumors 1.1 (RECIST 1.1) and modified Response Evaluation Criteria in Solid Tumors (mRECIST) based on liver dynamic CT or MRI (if appropriate) [19]. The objective response rate (ORR) was defined as the proportion of patients who achieved a complete response (CR) or partial response (PR), and the disease control rate (DCR) was defined as the proportion of patients who achieved CR, PR, or stable disease (SD). Furthermore, tumor markers serum alpha-fetoprotein (AFP) and protein induced by vitamin K absence or antagonist-II (PIVKA-II) were also assessed every 6 to 12 weeks to characterize the biological response. The safety assessment complied with the Common Terminology Criteria for Adverse Events version 5.0. Clinical outcomes, including OS (the time from the start date of systemic chemotherapy to death), PFS (the time from the start date of systemic chemotherapy to the date of disease progression or death from any cause), radiological response, and treatment-related AEs, were assessed.

### 2.3. Statistical Analysis

All data are expressed as the mean ± standard deviation, median (interquartile range [IQR]), and No. (%), as appropriate. Continuous variables were compared using the Student’s *t*-test (or Mann–Whitney test, if appropriate), and categorical variables were compared using the chi-square test or Fisher’s exact test. The Kaplan–Meier method was used to estimate the OS and PFS. Multivariate Cox regression analysis was performed to identify the independent prognostic factors.

Furthermore, to reduce the effect of selection bias and potential confounders between the two groups, propensity score (PS) matching with a 1:1 ratio and inverse probability of treatment weighting (IPTW) analyses were performed.

Statistical analyses were conducted using the SAS software (ver. 9.4; SAS Institute) and R software (version 4.0.2, http://cran.r-project.org/, accessed on 7 July 2021). Two-sided *p*-values < 0.05 were considered indicative of statistical significance.

## 3. Results

### 3.1. Baseline Characteristics

A total of 232 patients with unresectable HCC who were treated with LENV (*n* = 146) or ATE/BEV (*n* = 86) as first-line systemic treatment were included. The baseline characteristics of the two treatment groups are compared in Table 1. For the entire cohort, the mean age of patients was 62 years, and men were predominant (83.6%). HBV was the main etiology of HCC, accounting for 65.5% of all cases. According to the BCLC staging system, BCLC stage C was observed in 86.2% and BCLC B in 13.8% of patients. With regard to liver function, 90.0% and 10.0% of patients were classified into Child–Turcotte–Pugh (CTP) classes A and B, respectively. Solitary tumors or no intrahepatic lesion were observed in 32.3% of patients, and the median maximal diameter of tumors was 6.0 cm (IQR, 2.8–11.3 cm). Macroscopic vascular invasion and extrahepatic metastasis were detected in 55.1% and 51.3% of patients, respectively. The median serum AFP level was 159 ng/mL (IQR, 12–2698), and the median serum PIVKA-II level was 934 mAU/mL (IQR, 58–10,218). One hundred fifty-three (65.9%) patients received loco-regional treatment before the administration of first-line systemic treatment. The proportion of patients with previous surgery was significantly higher in ATE/BEV group (26.7% vs. 13.0%, *p* = 0.013), while the proportion of other previous loco-regional treatments was comparable in both groups. Among 60 patients who received radiation therapy, 59 patients received radiation to intrahepatic lesions, and only one patient in the ATE/BEVgroup received radiation to extrahepatic lesion.

The LENV group had a significantly higher proportion of patients with ECOG 0 performance status than the ATE/BEV group (71.9% vs. 41.9%, *p* < 0.001) (Table 1). However, ATE/BEV group patients had significantly more favorable outcomes compared to those from the LENV group in terms of BCLC stage (BCLC stage B, 20.9% vs. 9.6%, *p* = 0.001) and the presence of extrahepatic metastasis (43.0% vs. 62.3%, *p* = 0.006).

ECOG PS, Eastern Cooperative Oncology Group performance status; HBV, hepatitis B virus; HCV, hepatitis C virus; BCLC, Barcelona Clinic Liver Cancer; AFP, alpha-fetoprotein; PIVKA-II, protein induced by vitamin K absence or antagonist-II.

### 3.2. Treatment Responses

Treatment responses during treatment according to RECIST 1.1 and mRECIST criteria are described in Table 2. According to RECIST criteria 1.1, CR was observed in two (1.4%) patients in the LENV group and three (3.5%) patients in the ATE/BEV group. The ORR was similar between the LENV and ATE/BEV groups (31.5% vs. 32.6%; *p* = 0.868). The DCR was also similar between the LENV and ATE/BEV groups (76.7% vs. 75.6%; *p* = 0.760). Assessment of the treatment response according to mRECIST criteria also yielded comparable results, that is, the proportions of patients with CR, ORR, and DCR were not statistically different between the two treatment groups (all *p* > 0.05).

Treatment responses were assessed according to the etiology of HCC. In ATE/BEV group, the ORRs assessed by RECIST version 1.1 in patients with viral etiology and those without were 35.4% and 23.8%, respectively (*p* = 0.325), and patients with nonalcoholic fatty liver disease (NAFLD)-related HCC showed ORR of only 10.0%. Conversely, in the LENV group, the ORR assessed by RECIST version 1.1 in patients with viral etiology and those without were 31.5% and 33.3%, respectively (*p* = 0.836), and patients with NAFLD-related HCC showed ORR of 36.0%.

### 3.3. OS and PFS

**Figure 1:**
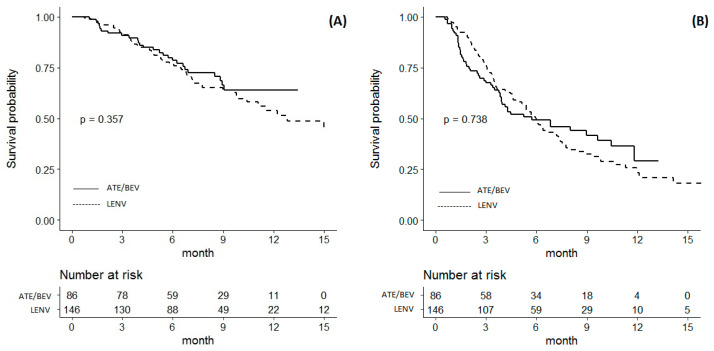
Kaplan–Meier analysis of survival outcomes in patients treated with atezolizumab plus bevacizumab versus lenvatinib. (**A**) Overall survival; (**B**) progression-free survival.

The median follow-up duration was 7.2 months in the LENV group and 7.7 months in the ATE/BEV group. A total of 53 patients (36.3%) died in the LENVA group, and 26 patients (30.2%) died in the ATE/BEV group during follow-up. The median OS was not significantly different between the LENV and ATE/BEV groups (12.8 [6.7–18.9] months vs. not reached; *p* = 0.357) (Figure 1A). A total of 84 patients (57.5%) in the LENV group and 47 patients (54.7%) in the ATE/BEVA group experienced disease progression. The median PFS was not significantly different between the LENV and ATE/BEV groups (6.0 [5.2–6.7] vs. 5.7 [2.1–9.3] months; *p* = 0.738) (Figure 1B).

**Figure 2:**
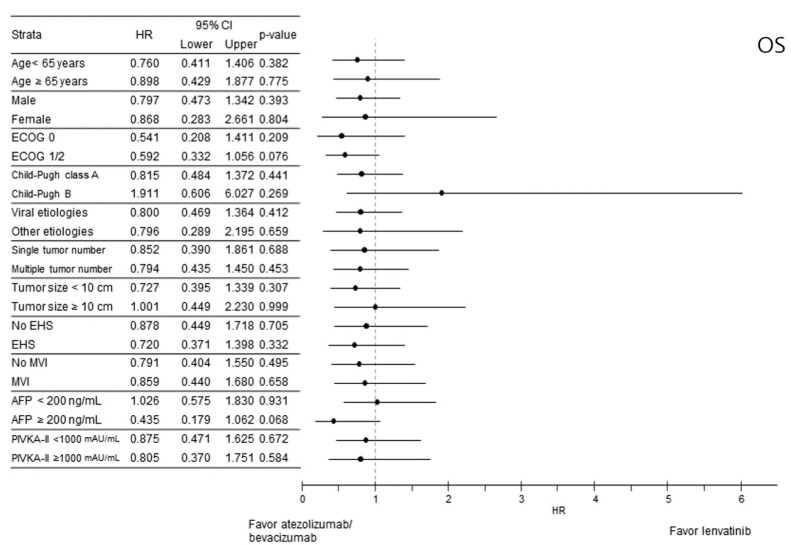
Forest plot of overall survival in subgroups of the entire cohort. Overall survival (OS).

**Figure 3:**
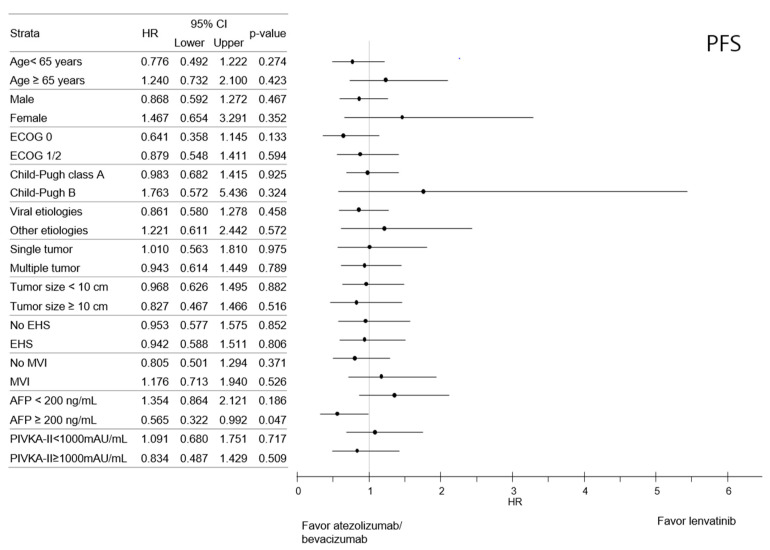
Forest plot of progression-free survival in subgroups of the entire cohort. Progression-free survival (PFS).

Subgroup analysis indicated that the OS was comparable between the LENV and ATE/BEV groups based on all strata (Figure 2). Likewise, the PFS between LENV and ATE/BEV patients was also comparable in most subgroups, except for patients with higher serum AFP (≥200 ng/mL). The ATE/BEV group had a better median PFS than the LENV group, with an HR of 0.565 (95% CI 0.322–0.992, *p* = 0.047) (Figure 3).

### 3.4. PS-Matched and IPTW Analyses of Clinical Efficacy

The PS was calculated using logistic regression, where variables of age, sex, tumor size (>10 cm vs. ≤10 cm), tumor number (single vs. multiple), extrahepatic metastasis, macrovascular invasion or regional lymph node involvement, platelet count (>100 × 10^3^/µL vs. ≤100 × 10^3^/µL), Child–Pugh score, AFP, and PIKVA-II were entered. The mean standardized differences were depicted in Supplementary Figure S2.

The 1:1 PS-matched analysis generated 78 pairs, and the baseline characteristics of the LENV and ATE/BEV groups are shown in Supplementary Table S1. The median OS of the LENV and ATE/BEV groups was not statistically different (median 19.9 [9.8–19.9] vs. not reached [9.1–N/A] months; *p* = 0.897) (Supplementary Figure S3A). The median PFS of the LENV and ATE/BEVA groups also was not statistically different (7.3 [5.7–9.4] vs. 5.7 [3.8–10.5] months, respectively; *p* = 0.391) (Supplementary Figure S3B). The ORR assessed by RECIST version 1.1 of LENV and ATE/BEV groups in the PS-matched cohort was statistically comparable (37.2% vs. 32.0%, *p* = 0.501, Table 3).

The baseline characteristics of the LENV and ATE/BEV groups after adjustment through IPTW analysis are shown in Supplementary Table S1. The median OS was not statistically different between the LENV and ATE/BEV groups (median 19.9 [11.1–N/A] vs. not reached [8.5–N/A] months, respectively; *p* = 0.508) (Supplementary Figure S3C). The median PFS also was not statistically different (6.0 [5.0–7.3] vs. 4.3 [3.0–8.0] months, respectively; *p* = 0.468) (Supplementary Figure S3D). The ORR assessed by RECIST version 1.1 in the IPTW-analyzed cohort was statistically comparable between the two treatment groups (32.4% vs. 31.4%, respectively, *p* = 0.861, Table 3).

When we analyzed PS-matched and IPTW analyses incorporating ECOG PS as well as the previously mentioned variables, similar results were reproduced. Using PS-matched analyses, neither OS (*p* = 0.644, Supplementary Figure S4A) nor PFS (*p* = 0.733, Supplementary Figure S4B) was statistically different between the two treatment groups. Likewise, using IPTW analyses, neither OS (*p* = 0.611, Supplementary Figure S4C) nor PFS (*p* = 0.755, Supplementary Figure S4D) was statistically different between the two treatment groups. In addition, the ORRs assessed by RECIST version 1.1 in the PS-matched (*p* = 0.853) and IPTW-analyzed (*p* = 0.822) cohorts were statistically comparable between the two treatment groups.

### 3.5. Subsequent Therapy

After disease progression, 27 patients from the ATE/BEV group received subsequent anticancer treatment: sorafenib (*n* = 17), lenvatinib (*n* = 8), or locoregional treatment (*n* = 2). In the LENV group, 51 patients received post-progression treatment after LENV: sorafenib (*n* = 29), locoregional treatment (*n* = 12), immune checkpoint inhibitor (*n* = 6), or cytotoxic chemotherapy ((*n* = 4)). The subsequent treatments are listed in Supplementary Table S2.

### 3.6. Safety

Treatment-related AEs of any grade occurred in 103 patients (70.5%) of the LENV group and 67 patients (77.9%) of the ATE/BEV group (*p* = 0.282). Grade ≥ 3 AEs occurred in 42.8% and 21.9% of patients in the ATE/BEV and LENV groups, respectively (*p* = 0.141). All AEs per treatment group are shown in Table 4. The most common AEs in the LENV group were anorexia (28.8%), fatigue (24.7%), and aminotransferase (AST) elevation (24.0%). The most common AEs in the ATE/BEV group were hypertension (41.9%), followed by AST elevation (37.2%), thrombocytopenia (36.0%), and fatigue (36.0%). All AEs were manageable with supportive care, and there were no grade 5 AEs directly causing death in either treatment group. AEs leading to the discontinuation of treatment occurred in 12 (8.2%) and 5 (5.8%) patients in the LENV and ATE/BEV groups, respectively (*p* = 0.486). The most common reasons for discontinuation of anticancer treatment were hepatic decompensation (*n* = 3) and gastrointestinal (GI) discomfort (*n* = 3) in the LENV group, whereas GI perforation (*n* = 3), GI bleeding (*n* = 1) and intracranial hemorrhage (*n* = 1) were most common in the ATE/BEV group.

## 4. Discussion

To the best of our knowledge, this is the first study to compare treatment efficacy and safety of ATE/BEV and LENV as first-line regimens for unresectable HCC. The two regimens exhibited comparable efficacy, and there was no statistical difference in OS and PFS between treatment groups in the unadjusted, PS-matched, and IPTW analyses. The current results are different from those of the IMbrave 150 study comparing ATE/BEV with SOR, wherein the PFS and OS curves of the ATE/BEV group were greater than those of the SOR group from the beginning, then meeting the co-primary endpoints of PFS and OS [15]. In terms of PFS, LENV showed a significant improvement of 7.4 months compared to SOR (3.7 months) in the phase III REFLECT trial [9]. Meanwhile, ATE/BEV resulted in a PFS of 6.8 months in the IMbrave 150 trial, suggesting that ATE/BEV and LENV regimens had comparable outcomes with regard to PFS, even though the comparison is made between two independent studies.

Although the median OS in the ATE/BEV group was not reached in our study, the survival curve of the ATE/BEV and LENV groups showed an overlapping trend to the current time point, without any significant difference. This result was unexpected, as the REFLECT trial [9] showed comparable OS between the LENV and SOR groups, whereas the survival curve of the ATE/BEV group exceeded that of the SOR group, showing early separation in the IMbrave150 study [15]. Considering the results of the two previous studies described above, the ATE/BEV group would have shown better OS compared to the LENV group in our study. In an attempt to better understand this unexpected outcome, we identified that 60% (51/84) of patients with progression in the LENV group received subsequent anticancer treatment, with 14% (12/84) receiving locoregional treatment such as transarterial chemoembolization to control intrahepatic HCC. The most important reason for the difference in subsequent therapy may come from the different timing of introduction of two drugs. LENV was first approved in August 2018 in South Korea, whereas ATE/BEV was first approved in June 2021. The subsequent treatment that accounts for the largest portion is the same, i.e., sorafenib in both LENV and ATE/BEV group (56.8% and 63.0%). However, other than sorafenib, patients in the ATE/BEV group mainly received LENV, while patients in the LENV group received various kinds of other existing treatment such as conventional chemotherapy or transarterial therapy, which might have been more effective overall. In the REFLECT trial [9], the OS of the LENV group was 13.6 months in the overall population, whereas the OS of the LENV group was 17.6 months in the Japanese subset [20]. The favorable OS in the Japanese subset was explained in part by the higher rate of up to 70% of patients in the Japanese subset receiving subsequent anticancer treatment. Collectively, as in the RELECT trial [9], effective subsequent treatment in the LENV group of our study may have contributed to the comparable early OS in the LENV group. Nonetheless, considering the long durable response (ATE/BEV vs. LENV group; 18.1 vs. 7.3 months) and the favorable long-term survival rate for ATE/BEV reported in the IMbrave 150 study [15], it is necessary to confirm the OS of both groups in the long-term follow-up setting.

Both ATE/BEV and LENV have been established as first-line therapy for advanced HCC, and there is controversy over which regimen should be selected as the initial treatment for patients with newly diagnosed advanced HCC. Subgroup analyses revealed that among patients with a baseline AFP level > 200 ng/mL, PFS was longer in the ATE/BEV group compared to the LENV group (11.8 vs. 5.5 months, *p* = 0.047) with HR 0.565 (95% CI 0.322–0.922). Except for the baseline AFP level, ATE/BEV and LENV showed comparable survival outcomes across baseline characteristics and tumor burden. Whether ATE/BEV is more beneficial in patients with AFP > 200 ng/mL should be confirmed in future studies.

In a recently published meta-analysis, immune-checkpoint inhibitor showed reduced efficacy in NAFLD-related HCC, probably owing to NAFLD-related aberrant T cell activation causing tissue damage leading to impaired immune surveillance [21]. In this study, ATE/BEV showed reduced efficacy in HCC without viral etiology compared to HCC with viral etiology(23.8% vs. 35.4%), and the ORR of ATE/BEV was particularly lower as 10.0% in NAFLD-related HCC patients. Thus, the trend toward lower treatment efficacy of immune-based treatment among non-viral HCC cases, especially for NAFLD-related HCC, was reproduced in a similar pattern. However, considering the small sample size of our study, elucidating whether the effects of immune-checkpoint inhibitor differ by etiology should be investigated in the future evaluations.

With regard to the frequency of overall and serious treatment-related AEs, there was no significant difference between the ATE/BEV and LENV groups. However, gastrointestinal perforation occurred in three patients (3.5%) of the ATE/BEV group, while only one patient (0.7%) experienced a perforation event in the LENV group. Although gastrointestinal perforation occurred in a small proportion of patients in both groups, the incidence of gastrointestinal perforation seemed to be higher in the ATE/BEV group. Gastrointestinal perforation is a serious AE associated with VEGF inhibitors [22]. Many patients with advanced HCC are exposed to local treatments, such as radiation and transarterial radioembolization (TARE), which may act as predisposing factors for gastrointestinal perforation. In our study, two patients with gastrointestinal perforation had previously received radiotherapy and TARE, respectively. The incidence of gastrointestinal bleeding was not significantly different between the two treatment groups. However, intracranial hemorrhage occurred in one patient of the ATE/BEV group. Therefore, awareness of possible bleeding and monitoring is warranted for patients with HCC undergoing treatment with ATE/BEV [23].

Our study had several limitations. First, since the study design was retrospective and the ATE/BEV combination regimen had been approved in Korea since July 2020, the follow-up duration was not sufficient for assessing mortality, which may introduce selection bias, particularly with regard to treatment allocation. To overcome this, various statistical adjustments, including PS-matched and IPTW analyses based on real-world experience from three independent academic hospitals, were performed. Comparable results were obtained after adjustment. Nevertheless, long-term follow-up studies with data on various salvage regimens after progression are required to corroborate real-world evidence for guiding treatment strategies.

## 5. Conclusions

In conclusion, ATE/BEV and LENV treatment exhibited comparable real-world efficacy and safety as a first-line therapy for the treatment of unresectable HCC. Further studies with a larger sample size and longer follow-up are needed to validate these results.

## Figures and Tables

**Table 1:** Baseline characteristics of the patients.

Variable	All (*n* = 232)	LENV Group (*n* = 146)	ATE/BEV Group (*n* = 86)	*p*-Value
Age, years	62 (56–62)	62 (55–70)	62 (56–71)	0.564
Male	194 (83.6)	124 (84.9)	70 (81.4)	0.582
ECOG PS				
0	141 (60.8)	105 (71.9)	36 (41.9)	<0.001
1/2	91 (39.2)	41 (28.1)	50 (58.2)	
Etiology				0.582
HBV	152 (65.5)	90 (61.6)	62 (72.1)	
HCV	22 (9.5)	19 (13.0)	3 (2.5)	
Alcohol	23 (9.9)	12 (8.2)	11 (12.8)	
Others	35 (15.1)	25 (17.1)	10 (11.7)	
BCLC stage				
B	32 (13.8)	14 (9.6)	18 (20.9)	0.001
C	200 (86.2)	132 (90.4)	68 (79.1)	
Child-Pugh Class				
A	209 (90.0)	127 (87.0)	82 (95.4)	0.169
B	23 (10.0)	19 (13.0)	4 (4.7)	
Number of intraheptic tumors				
0	20 (8.6)	14 (9.6)	6 (7.0)	0.001
1	55 (23.7)	34 (23.3)	21 (24.4)	
2	41 (17.7)	14 (9.6)	27 (31.4)	
3	18 (7.8)	12 (8.2)	6 (7.0)	
>3	98 (42.2)	72 (49.3)	26 (30.2)	
Maximal size of intraheptic tumor, cm	6.0 (2.8–11.3)	7.0 (3.0–11.6)	4.9 (2.2–10.0)	0.197
Extrahepatic metastasis	128 (55.1)	91 (62.3)	37 (43.0)	0.006
Macrovascular invasion	119 (51.3)	76 (52.1)	43 (50.0)	0.787
Platelet count > 100 × 10^3^/uL	172 (74.1)	105 (71.9)	67 (77.9)	0.314
AFP, ng/mL	159 (12–2698)	185 (12–3138)	91 (12–2383)	0.303
PIVKA-II, mAU/mL	934 (58–10,218)	1899 (75–12,965)	223 (38–4872)	0.540
Previous treatment	153 (65.9)	93 (63.7)	60 (69.8)	0.391
Surgery	42 (18.1)	19 (13.0)	23 (26.7)	0.013
Transarterial therapy	122 (52.6)	77 (52.7)	45 (52.3)	1.000
Radioablation therapy	28 (12.1)	19 (13.0)	9 (10.5)	0.678
Radiation therapy	60 (25.8)	40 (27.4)	20 (23.3)	0.298
Presence of varices	147 (63.4)	87 (59.6)	60 (69.8)	0.295
Treated varices at baseline	48 (20.7)	28 (19.2)	20 (23.3)	0.195

Values are presented as median (interquartile range) or number (%).

**Table 2:** Clinical response of patients during treatment.

Response	RECIST 1.1				Modified RECIST	
	LENV Group	ATE/BEV Group	*p*-Value	LENV Group	ATE/BEV Group	*p*-Value
Complete response	2 (1.4)	3 (3.5)	0.745	2 (1.4)	3 (3.5)	0.742
Partial response	44 (30.1)	25 (29.1)		47 (32.2)	27 (31.4)	
Stable disease	66 (45.2)	37 (43.0)		63 (43.2)	35 (40.7)	
Progressive disease	34 (23.3)	21 (24.4)		34 (23.3)	21 (24.4)	
Objective response rate	31.5	32.6	0.868	33.6	34.9	0.485
Disease control rate	76.7	75.6	0.760	76.8	75.6	0.682

Data are presented as *n* (%) or %, as appropriate. RECIST, Response Evaluation Criteria in Solid Tumors.

**Table 3:** Clinical responses and survival outcomes of atezolizumab plus bevacizumab vs. lenvatinib treatment groups.

Clinical Responses and Survival Outcomes	LENV Group	ATE/BEV Group	*p*-Value
**Entire cohort, unadjusted**	*n* = 146	*n* = 86	
Objective response rate, %	31.5	32.6	0.704
Overall survival, months (95% CI)	12.8 (6.7–18.9)	N/A	0.357
Progression-free survival, months (95% CI)	6.0 (5.2–6.7)	5.7 (2.1–9.3)	0.738
**Entire cohort, PS-matched**	*n* = 78	*n* = 78	
Objective response rate, %	37.2	32.0	0.501
Overall survival, month (95% CI)	19.9 (9.8–19.9)	N/A	0.897
Progression-free survival, months (95% CI)	7.3 (5.7–9.4)	5.7 (3.8–10.5)	0.391
**Entire cohort, IPTW analysis**	*n* = 136	*n* = 136	
Objective response rate, %	32.4	31.4	0.861
Overall survival, months (95% CI)	19.9 (11.1–N/A)	N/A	0.508
Progression-free survival, months (95% CI)	6.0 (5.0–7.3)	4.3 (3.0–8.0)	0.468

Objective response rate was assessed with RECIST 1.1.

**Table 4:** Adverse events.

	LENV Group (*n* = 146)		ATE/BEV Group (*n* = 86)	
Adverse Events	Any Grade *n* (%)	Grade 3 or 4 *n* (%)	Any Grade *n* (%)	Grade 3 or 4 *n* (%)
Hypertension	22 (15.1)	4 (2.7)	36 (41.9)	5 (5.8)
AST elevation	35 (24.0)	1 (0.7)	32 (37.2)	7 (8.1)
Thrombocytopenia	22 (15.1)	0	31 (36.0)	3 (3.5)
Fatigue	36 (24.7)	3 (2.1)	31 (36.0)	0
Anemia	8 (5.5)	1 (0.7)	22 (25.6)	1 (1.2)
Anorexia	42 (28.8)	10 (6.8)	20 (23.3)	0
ALT elevation	29 (19.9)	0	19 (22.1)	2 (2.3)
Proteinuria	25 (17.1)	4 (2.7)	19 (22.1)	1 (1.2)
Total bilirubin elevation	22 (15.1)	1 (0.7)	15 (17.4)	4 (4.7)
Nausea	14 (9.6)	0	11 (12.8)	0
Rash	2 (1.4)	0	10 (11.6)	2 (2.3)
Neutropenia	12 (8.2)	0	10 (11.6)	2 (2.3)
Pruritus	4 (2.8)	0	7 (8.1)	1 (1.2)
Gastrointestinal bleeding	4 (2.7)	4 (2.7)	5 (5.8)	3 (3.5)
Vomiting	6 (4.1)	0	4 (4.7)	0
Gastrointestinal perforation	1 (0.7)	1 (0.7)	3 (3.5)	3 (3.5)
Diarrhea	20 (13.7)	4 (2.7)	3 (3.5)	0
Hypothyroidism	12 (8.2)	0	2 (2.3)	0
Intracranial hemorrhage	0	0	1 (1.2)	1 (1.2)
Pulmonary embolism	1 (0.7)	1 (0.7)	1 (1.2)	1 (1.2)

AST, aspartate aminotransferase; ALT, alanine aminotransferase.

## Data Availability

The data presented in this study are available on request from the corresponding author.

## Supplementary Materials

The following supporting information can be downloaded at: https://www.mdpi.com/article/10.3390/cancers14071747/s1, Figure S1: Patient enrollment; Figure S2: Standardized mean differences through PS-based adjustment; Figure S3: Kaplan–Meier analysis of survival outcomes between the ATE/BEV and LENV groups after adjustment; Figure S4: Kaplan–Meier analysis of survival outcomes between the ATE/BEV and LENV groups incorporating ECOG PS with other covariates; Table S1: Baseline characteristics after adjustment; Table S2: Subsequent anticancer treatment.
