# Supplementary material for: Atezolizumab/Bevacizumab vs. Lenvatinib as First-Line Therapy for Unresectable Hepatocellular Carcinoma: A Real-World, Multi-Center Study
Source: Cancers (Basel). 2022 Mar 29;14(7):1747. doi: 10.3390/cancers14071747 (PMC8996911; doi:10.3390/cancers14071747)
Supplement: Supplementary file 1 [file cancers-14-01747-s001.zip › cancers-1634252-supplementary.pdf]

Supplementary Materials

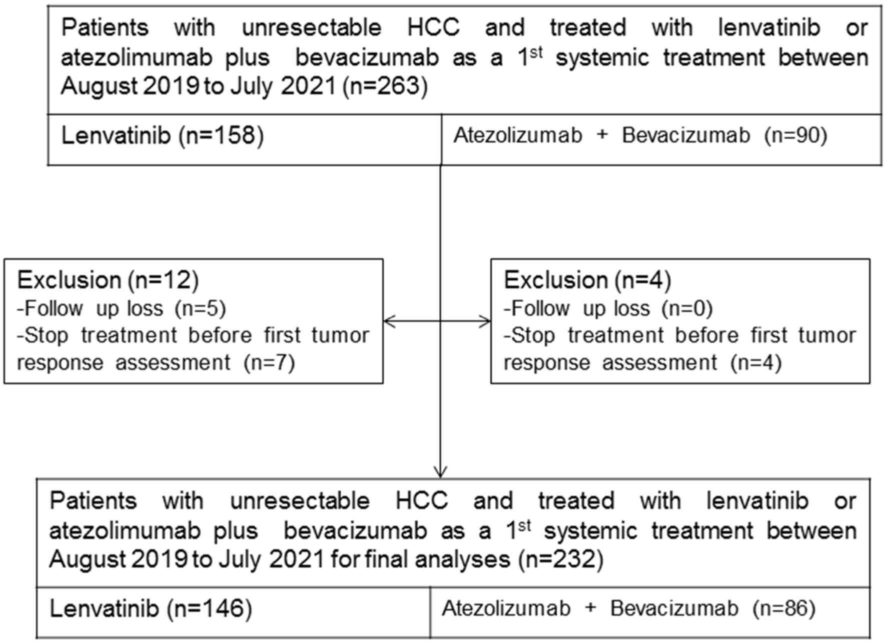

Figure S1. Patient enrollment.

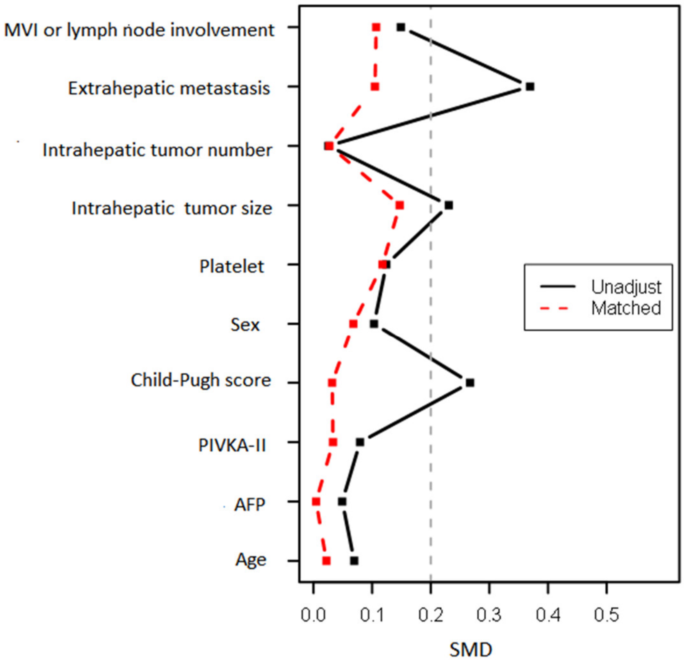

Figure S2. Standardized mean differences through PS-based adjustment.

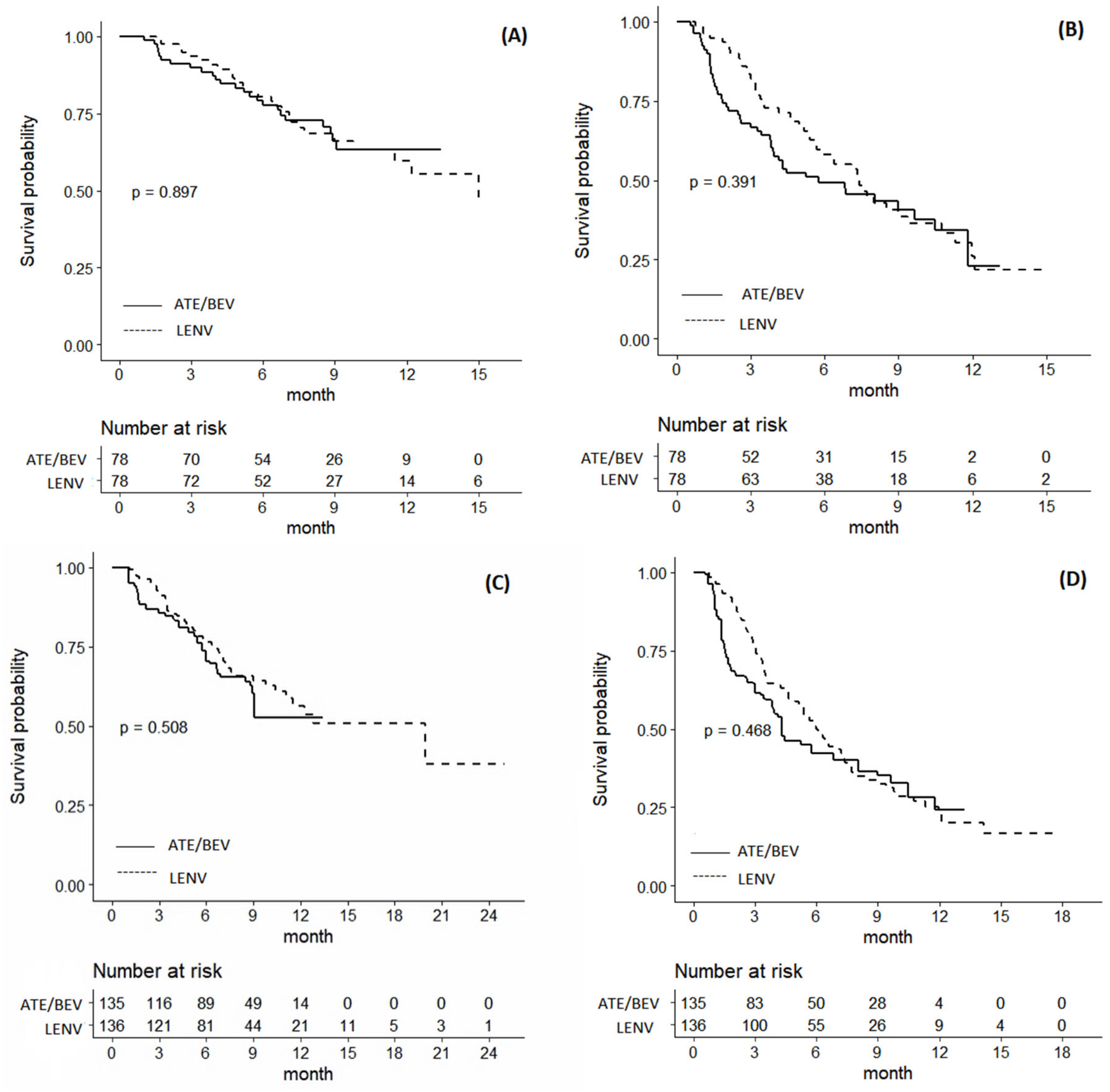

Figure S3. Kaplan–Meier analysis of survival outcomes between the ATE/BEV and LENV groups after adjustment: (**A**) Overall survival by PS-matched analysis, (**B**) Progression-free survival by PS-matched analysis, (**C**) Overall survival by IPTW analysis, and (**D**) Progression-free survival by IPTW analysis.

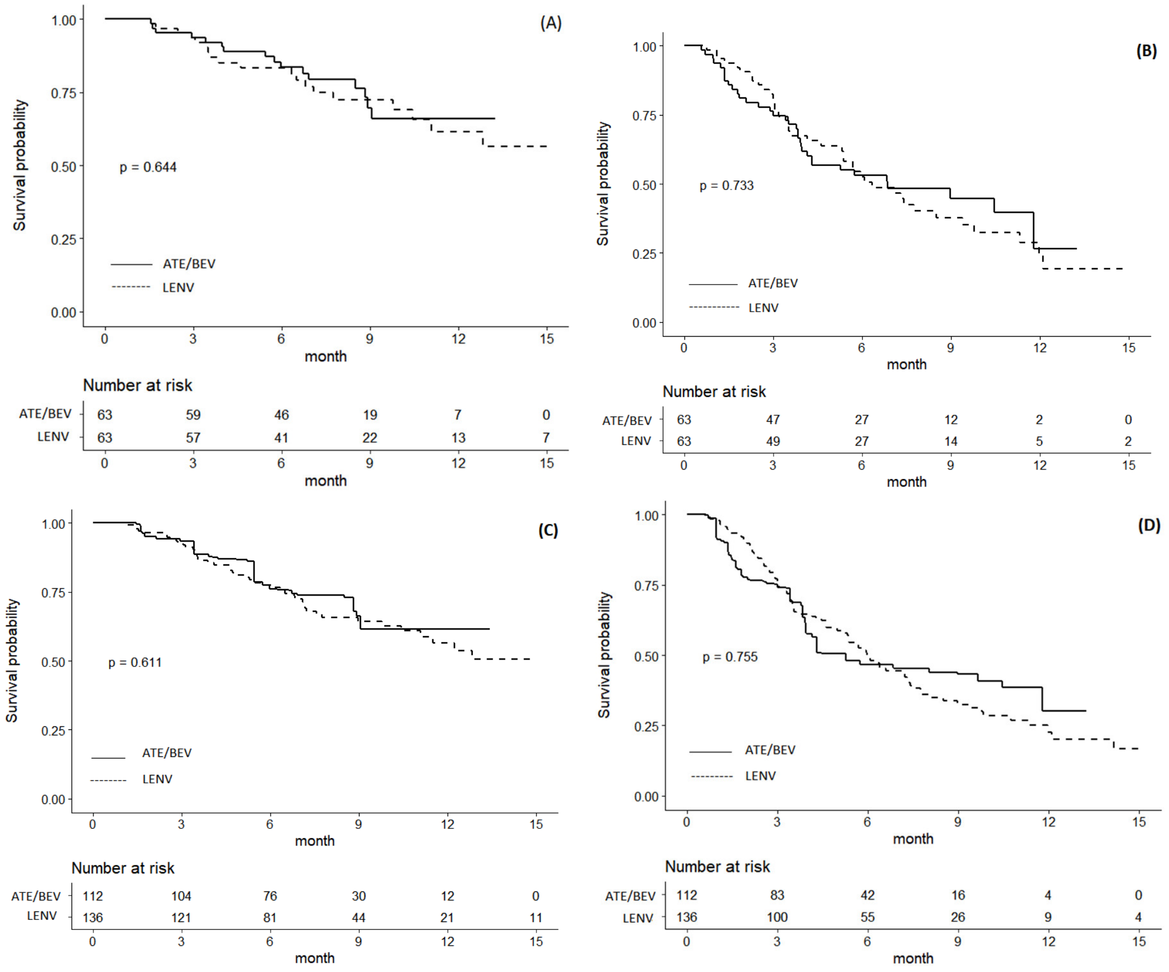

Figure S4. Kaplan–Meier analysis of survival outcomes between the ATE/BEV and LENV groups incorporating ECOG PS with other covariates: (**A**) Overall survival for PS-matched analysis, (**B**) Progression-free survival for PS-matched analysis, (**C**) Overall survival by IPTW analysis, and (**D**) Progression-free survival by IPTW analysis.

Table S1. Baseline characteristics after adjustment.

Variable	PS-Matched			IPTW Analysis		
	LENV Group (n = 78)	ATE/BEV Group (n = 78)	*P*-Value	LENV Group	ATE/BEV Group	*P*-Value
Age, years	62 (55–71)	62 (56–71)	0.894	62 (55–70)	62 (57–71)	0.508
Male	65 (83.33)	67 (85.9)	0.657	115 (84.6)	116 (86.2)	0.706
Child-Pugh score	5 (5–5)	5 (5–5)	0.429	5 (5–6)	5 (5–5)	0.018
Multiple intra-hepatic tumors (vs. single)	51 (65.4)	52 (66.7)	0.866	91 (66.9)	93 (69.0)	0.712
Intra-hepatic tumor >10 cm	22 (28.2)	17 (21.8)	0.355	44 (32.4)	42 (3.01)	0.811
Extrahepatic metastasis	40 (51.3)	36 (46.2)	0.522	86 (63.2)	81 (59.8)	0.565
MVI or regional lymph node involvement	53 (68.0)	49 (62.8)	0.501	92 (67.7)	95 (69.9)	0.692
Platelet count >100x10^3/ul (vs. ≤ 100 ×10^3/μL)	55 (70.5)	59 (75.6)	0.470	97 (71.3)	92 (67.9)	0.539
AFP, ng/mL	92 (10–1398)	70 (11–2553)	0.939	185 (13–2994)	92(12–2327)	0.416
PIVKA-II, mAU/mL	1206 (58–9950)	367 (38–5120)	0.168	1900 (77–12923)	224 (39–46259)	0.007

PS, propensity score; IPTW, inverse probability of treatment weighting; MVI, macrovascular invasion; AFP, alpha-fetoprotein; PIVKA-II, protein induced by vitamin K absence or antagonist-II.

Table S2. Subsequent anticancer treatment.

	LENV Group (n = 146)	ATE/BEV Group (n = 86)
No. of patients who experienced disease progression	84	47
No. of patients who received subsequent anticancer treatment	51	27
Sorafenib	29	17
Lenvatinib	-	8
Cabozantinib	0	0
Conventional chemotherapy	4	0
Immune checkpoint inhibitor	6	0
Locoregional treatment	12	2
